# Associations between social networks, messaging apps, addictive behaviors, and sleep problems in adolescents: the EHDLA study

**DOI:** 10.3389/fnbeh.2025.1512535

**Published:** 2025-01-24

**Authors:** María Navalón-González, José Adrián Montenegro-Espinosa, Héctor Gutiérrez-Espinoza, Jorge Olivares-Arancibia, Rodrigo Yañéz-Sepúlveda, Daniel Duclos-Bastías, Miriam Garrido-Miguel, Arthur Eumann Mesas, José Francisco López-Gil, Estela Jiménez-López

**Affiliations:** ^1^Health and Social Research Center, Universidad de Castilla-La Mancha, Cuenca, Spain; ^2^One Health Research Group, Universidad de las Américas, Quito, Ecuador; ^3^Faculty of Education, Universidad Autónoma de Chile, Santiago, Chile; ^4^AFySE Group, Research in Physical Activity and School Health, School of Physical Education, Faculty of Education, Universidad de las Américas, Santiago, Chile; ^5^Faculty Education and Social Sciences, Universidad Andres Bello, Viña del Mar, Chile; ^6^iGEO Group, School of Physical Education, Pontificia Universidad Católica de Valparaíso, Valparaíso, Chile; ^7^IGOID Research Group, Faculty of Sport Sciences, University of Castilla-La Mancha, Toledo, Spain; ^8^Research Network on Chronicity, Primary Care and Health Promotion (RICAPPS), Cuenca, Spain; ^9^Faculty of Nursing, Universidad de Castilla-La Mancha, Albacete, Spain; ^10^Centro de Investigación Biomédica en Red de salud Mental, Instituto de Salud Carlos III, Madrid, Spain

**Keywords:** social network, social media addiction, addictive behavior, sleep, teenagers

## Abstract

**Objective:**

The current study aims to provide a comprehensive analysis of the relationships between social network (SN) use, messaging apps use, and addictive behaviors related to SNs, and sleep-related problems in a sample of Spanish adolescents.

**Methods:**

This was a cross-sectional study using data from the Eating Healthy and Daily Life Activities (EHDLA) project, which involved adolescents aged 12–17 years from three secondary schools in *Valle de Ricote* (Region of Murcia, Spain). A sample of 632 adolescents was studied. The use of SN (i.e., Facebook, Twitter, Instagram, Snapchat or TikTok) or messaging applications (i.e., WhatsApp) was assessed via a scale including one item for each SN, in which adolescents were asked what type of SN they used and the usage profile of each SN. The Short Social Networks Addiction Scale-6 Symptoms (SNAddS-6S) was used to determine SN addictive behaviors. Generalized linear regression analyses with a negative binomial distribution were performed to determine the associations of SN use or SN addictive behaviors with sleep-related problems. These analyses were adjusted for age, sex, body mass index, socioeconomic level, physical activity, sedentary behavior, and adherence to the Mediterranean diet.

**Results:**

Higher SN use was related to greater presence of sleep-related problems [prevalence ratio (PR) = 1.04; 95% confidence interval (CI) 1.01–1.07; *p* = 0.015]. Additionally, the higher the score on the addictive behaviors toward SN use scale was, the more sleep-related problems were identified (PR = 1.15; 95% Cl 1.09 to 1.21; *p* < 0.001). Specifically, only the use of Twitter was significantly associated with sleep-related problems (PR = 1.10; 95% Cl 1.01 to 1.21; *p* = 0.035). In terms of addictive behaviors related to SN use, mood modification, relapse, withdrawal, and conflict were significantly associated with sleep-related problems (mood modification: PR = 1.58; 95% CI 1.36 to 1.84; *p* < 0.001; relapse: PR = 1.24; 95% CI 1.07 to 1.43; *p* = 0.004; withdrawal: PR = 1.28; 95% CI 1.08 to 1.51; *p* = 0.004; conflict: PR = 1.19; 95% CI 1.01 to 1.39; *p* = 0.037).

**Conclusion:**

Our results suggest a relationship between SN use, SN addictive behaviors, and sleep-related problems in adolescents. These cross-sectional results should be confirmed in longitudinal and intervention studies.

## 1 Introduction

Sleep is fundamental to all stages of life (Ohayon et al., 2004) as a factor essential for physical health (Medic et al., 2017) and mental health (Palmer et al., 2024). Healthy sleep is defined by an adequate duration, high quality, appropriate timing, and consistency, as well as by the absence of sleep disturbances and disorders (Watson et al., 2015). Sleep-related problems present themselves in a variety of ways and generally fall into different categories that affect both the quality and quantity of sleep (Sadeh et al., 2011). Sleep-related problems have been associated with consequences, such as increased stress response, emotional distress, mood disorders, cognitive deficits, and reduced performance and quality of life (Medic et al., 2017).

It has been suggested that sleep-related problems can have especially deleterious consequences in adolescence, since this period is characterized by physical, cognitive, and emotional transformations (Committee on the Neurobiological and Socio-behavioral Science of Adolescent Development and Its Applications, Board on Children, Youth, and Families, Division of Behavioral and Social Sciences and Education, Health and Medicine Division, and National Academies of Sciences, Engineering, and Medicine, 2019). Sleep-related problems are very common in adolescents, with variable prevalence rates ranging between 10 and 40%, and are more frequent in girls (Ceruelo and Marcos, 2018). Furthermore, chronic sleep loss in adolescents has been related to short- and long-term consequences, such as drowsiness, daytime disturbances, depression, increased risk of obesity, and increased rates of traffic accidents due to sleepiness (Owens et al., 2014).

In recent years, screen-based activities, including social network (SN) use, have been highlighted as a significant factor contributing to delays in bedtime among young people, leading to increased sleep deprivation (LeBourgeois et al., 2017). Specifically, a systematic review by Alonzo et al. (2021), which examined the results of 36 cross-sectional studies and six prospective cohort studies, revealed that excessive social media use could be related to poor sleep quality (and negative mental health) in youth (aged 16–25 years). Fear of missing out can contribute to shorter sleep duration in adolescents by driving late-night social media use and increasing pre-sleep cognitive arousal (Scott and Woods, 2018). However, while the association between SN use and sleep problems in adolescents has been studied extensively, the relationship between addictive behaviors toward SN use and these problems has been less explored.

In Spain, 80% of the population is already on, at least, one SN (We Are Social, 2021). On the basis of official data from the Spanish government (Instituto Nacional de Estadística, 2019) in 2023, 70.6% of children and adolescents aged 10–15 years had a cell phone, 93.1% had been computer users in the previous 3 months, and 94.7% had been internet users in the previous 3 months. Likewise, according to a report among Spanish children and adolescents, 91% of this population between 8 and 17 years of age access SNs on a regular basis and make intensive consumption, especially on weekends, with uses ranging from more than 3 h a day from Monday to Friday to more than 5 h a day on Saturdays and Sundays (Carat, 2022). In addition, it has been reported that parents or guardians often do not know how much their children use SN, with 75% of parents stating that their children connect to the internet daily, whereas 86.4% of the children themselves acknowledge using the internet more frequently (Carat, 2022). Moreover, another report noted that the most commonly used SN is Instagram (47.7% of children under 15 years of age use this), with TikTok ranking second, with a 37.7% (Fernández, 2022).

Given this context, the current study aims to provide a comprehensive analysis of the relationships between SN use, messaging apps use, and addictive behaviors related to SNs, and sleep-related problems in a sample of Spanish adolescents. We hypothesized that higher levels of SN use and addictive behaviors toward their use would be significantly associated with increased sleep-related problems. This study could inform interventions aimed at reducing SN use and mitigating its negative impacts on adolescent sleep health.

## 2 Methods

### 2.1 Population sample and study design

A cross-sectional study was conducted using data from the Eating Healthy and Daily Life Activities (EHDLA) project (López-Gil, 2022). The EHDLA study used a simple random sampling technique, with a minimum sample size of 1,138 participants. The sample consisted of adolescents aged 12–17 years from three secondary schools in the *Valle de Ricote*, Region of Murcia (Spain), during 2021–2022. In the present work, a secondary sample was used that included a total of 632 participants (57.0% girls), who presented complete data on all the variables of interest.

The inclusion criteria for the participants were (a) being between 12 and 17 years old and (b) residing in the *Valle de Ricote*. The exclusion criteria were (a) being exempt from the physical education subject at the school, (b) suffering from any pathology that contraindicated physical activity or required special attention, (c) being under any pharmacological treatment, (d) not being authorized by parents or legal guardians to participate in the research project, and (e) not agreeing to participate in the research project.

The parents or legal guardians of the adolescents received and signed an informed consent form to allow participation. They also received an information sheet with the objectives of the project, the tests and the questionnaires to be administered. In addition, the adolescents were asked about their willingness to participate. Data collection was carried out in physical education classes. Approval for the EHDLA project was obtained from the Bioethics Committee of the *Universidad de Murcia* (ID 2218/2018) and from the Ethics Committee of the *Complejo Hospitalario Universitario de Albacete* and the *Gestión Asistencial Integrada de Albacete* (ID 2021–86). In addition, it has been carried out following the ethical principles established in the Declaration of Helsinki of the World Medical Association, which provides guidelines for research with human beings, guaranteeing respect for the individual and his or her right to make informed decisions.

### 2.2 Instruments

#### 2.2.1 SN use

SN use (e.g., Facebook, Twitter, Instagram, Snapchat or TikTok) was evaluated via a scale including one item for each SN, in which adolescents were asked what type of SN they used and the usage profile of each SN, on the basis of five possible responses: (a) I never or rarely use them (0 points), (b) I am a low consumer (1 point), (c) I am a medium consumer (2 points), (d) I am a fairly high consumer (3 points), or (e) I am a very high consumer (4 points) (Casas y Madorell, 2007). The total of all SN responses was summed to calculate the SN use score (ranging from 0 to 20), with higher scores indicating greater SN use.

#### 2.2.2 Messaging application use

The use of the most popular messaging application (i.e., WhatsApp) (Dixon, 2024) was assessed via a single-item scale based on five possible responses ranging from “never” to “high” (0–4 points). Higher scores indicate longer usage times.

#### 2.2.3 Addictive behaviors toward SN use

The Short Social Networks Addiction Scale-6 Symptoms (SNAddS-6S) was used to determine addictive behaviors toward SN use. This scale includes six items reflecting the six basic symptoms of addictive behaviors (Griffiths, 2005), including tolerance (i.e., desire to use SNs more and more), withdrawal (i.e., presence of psychological and physical symptoms when unable to use SNs), salience (i.e., SN use becomes the highest priority concern and motivation), mood modification (i.e., changes in mood, due to relaxation or excitement associated with the use of SNs), conflict (i.e., impairment in other social and normal activities due to spending too much time on SNs) and relapse (i.e., when SN use is initially controlled, but subsequent use leads to a relapse). It also has a unifactorial structure (Cuadrado et al., 2020). In the absence of a validated cutoff point for the SNAddS-6S, the number of addictive behaviors related to SN use was treated as a continuous variable (ranging from 0–6).

#### 2.2.4 Sleep-related problems

Sleep-related problems were assessed via the BEARS scale (B = Bedtime Issues, E = Excessive Daytime Sleepiness, A = Night Awakenings, R = Regularity and Duration of Sleep, S = Snoring). This screening tool was designed to detect common sleep problems in the young population (aged 2–18 years) during a clinical interview (Owens and Dalzell, 2005). The scale consists of a series of items covering different sleep-related issues: difficulties at bedtime (e.g., trouble lying down or falling asleep), excessive daytime sleepiness (e.g., behaviors indicating sleepiness during the day), night awakenings, sleep regularity and duration, and snoring. A previous study demonstrated the concurrent validity of the Spanish translation of the BEARS scale for detecting sleep problems in children and adolescents aged 2–16 years (Bastida-Pozuelo and Sánchez-Ortuño, 2016). The responses are scored on a scale from 0 to 1, where 0 indicates the absence of the problem. The total score ranges from 0 to 5, with higher scores reflecting more severe sleep-related problems.

#### 2.2.5 Covariates

Sociodemographic factors such as biological sex and date of birth were self-reported. Socioeconomic status was assessed via the Family Affluence Scale (FAS-III) (Currie et al., 2008). The FAS-III score is calculated by summing the responses to 6 items related to household wealth and amenities: vehicle ownership, own bedroom, number of computers, number of bathrooms, dishwasher ownership, and number of vacations abroad. The total score is the sum of all responses and ranges from 0 to 13 points. Adolescent body weight was measured with an electronic scale (accurate to 0.1 kg) (Tanita BC-545, Tokyo, Japan), and height was determined with a portable height rod accurate to 0.1 cm (Leicester Tanita HR 001, Tokyo, Japan). Body mass index (BMI) was calculated by dividing body weight (in kg) by height (in meters squared). The Spanish version of the Youth Activity Profile (YAP-S) (Segura-Díaz et al., 2021), which was validated for children and adolescents, was used to evaluate physical activity and sedentary behavior. This is a 15-item self-administered questionnaire for adolescents aged 8–17; these variables were assessed during the past 7 days via a 5-point Likert scale. It includes three sections: (1) in-school activity (e.g., transportation, physical education), (2) out-of-school activity (e.g., before and after school, weekends), and (3) sedentary habits (e.g., TV, video games, computer, phone use). The Mediterranean Diet Quality Index for Children and Adolescents (KIDMED) (Serra-Majem et al., 2004) was used to assess adherence to the Mediterranean diet. This index ranges from −4 to 12 and is based on a 16-question tool that explores various aspects of the Mediterranean diet. Each affirmative response related to adherence to the Mediterranean diet is scored +1; conversely, negative responses related to adherence to the Mediterranean diet are scored −1.

#### 2.2.6 Justification for including covariates

The relationship between SN use or addictive behaviors toward SN use and sleep-related problems in adolescents could be influenced by several factors. The covariates included in this study—such as sex, age, socioeconomic status, physical activity, sedentary behavior, adherence to the Mediterranean diet, and BMI—are well-established factors related to sleep-related problems (Godos et al., 2024; Kline et al., 2021; Guo et al., 2014; Felden et al., 2015; Yang et al., 2017; Geva et al., 2020).

### 2.3 Statistical analysis

For sample characterization, data are expressed as medians and interquartile ranges (IQRs) for continuous variables and percentages for categorical variables. Generalized linear regression analyses with a negative binomial distribution were performed to determine the associations of SN use or SN addictive behaviors with sleep-related problems. The dispersion of the data was verified using the Cameron and Trivedi test, which indicated significant overdispersion. This means that the variance of the dependent variable was greater than its mean, suggesting that a negative binomial model is more appropriate than a Poisson model. Since the interaction term was not statistically significant, with a *p*-value of 0.623, boys and girls were analyzed together to increase the statistical power of the analyses conducted. Data were expressed as prevalence ratios (PRs), 95% confidence intervals (95% CIs), and *p*-values. Age, sex, socioeconomic status, physical activity, sedentary behavior, BMI, and adherence to the Mediterranean diet were considered covariates. All the statistical analyses were conducted via R software (Version 4.3.2) (R Core Team, Vienna, Austria) and RStudio (2023.09.1 + 494) (Posit, Boston, MA, USA). A *p*-value less than 0.05 was regarded as statistically significant.

## 3 Results

Table 1 shows the descriptive data of the study participants. The sample consisted of 632 participants. The median age of the sample was 14 years (IQR = 2.0). The median FAS-III score was 8 points (IQR = 2.0). Regarding the independent variables, the sample presented a median SN use of 13.0 points (IQR = 5.0). The median SNAddS-6S score was 2.0 (IQR = 2.0) (on a scale ranging from none to six behaviors). In relation to the presence of symptoms of addictive behaviors, the most frequently reported was “salience” (i.e., SN use becomes the highest priority concern and motivation) (63.3%). In contrast, the least reported symptom was “conflict” (22.9%). For sleep-related problems, the median BEARS scale score was 1.0 (IQR = 2.0).

**Table 1:** Descriptive data of study participants (*N* = 632).

Variables		Total
Age (years)	Median (IQR) [range]	14.0 (2.0) [12 to 17]
Sex	Boys (%)	272 (43.0)
	Girls (%)	360 (57.0)
FAS-III (score)	Median (IQR) [range]	8.0 (2.0) [1 to 13]
YAP-S (physical activity)	Median (IQR) [range]	2.6 (0.8) [1 to 13]
YAPS (sedentary behaviors)	Median (IQR) [range]	2.6 (0.8) [1 to 13]
BMI (kg/m ^2^)	Median (IQR) [range]	21.7 (5.7) [13.0 to 44.5]
KIDMED (score)	Median (IQR) [range]	7.0 (3.0) [−1 to 12]
SN use		
Instagram use (score)	Median (IQR) [range]	3.0 (2.0) [0 to 4]
TikTok use (score)	Median (IQR) [range]	3.0 (3.0) [0 to 4]
Twitter use (score)	Median (IQR) [range]	0.0 (1.0) [0 to 4]
Facebook use (score)	Median (IQR) [range]	0.0 (0.0) [0 to 4]
Snapchat use (score)	Median (IQR) [range]	0.0 (1.0) [0 to 4]
Social network use (score) †	Median (IQR) [range]	13.0 (5.0) [0 to 18]
Messaging application use		
WhatsApp use (score)	Median (IQR) [range]	2.5 (1.0) [0 to 4]
Addictive behaviors toward social network use		
Salience	No (%)	232 (36.7)
	Yes (%)	400 (63.3)
Tolerance	No (%)	480 (75.9)
	Yes (%)	152 (24.1)
Mood modification	No (%)	342 (54.1)
	Yes (%)	290 (45.9)
Withdrawal	No (%)	412 (65.2)
	Yes (%)	220 (34.8)
Relapse	No (%)	481 (76.1)
	Yes (%)	151 (23.9)
Conflict	No (%)	487 (77.1)
	Yes (%)	145 (22.9)
SNAddS-6S (score)	Median (IQR) [range]	2.0 (2.0) [0 to 6]
Sleep-related problems		
BEARS (score)	Median (IQR) [range]	1.0 (2.0) [0 to 5]

BEARS: B, bedtime issues; E, excessive daytime sleepiness; A, night awakenings; R, regularity and duration of sleep; S, snoring; FAS-III; the family affluence scale; BMI, body mass index; KIDMED, mediterranean diet quality index for children and teenagers; IQR, interquartile range; SNAddS-6S, Social Networks Addiction Scale-6 Symptoms; YAP-S, Spanish Youth Active Profile. † Sum of Instagram, TikTok, Twitter, Facebook, and Snapchat use.

**Figure 1:**
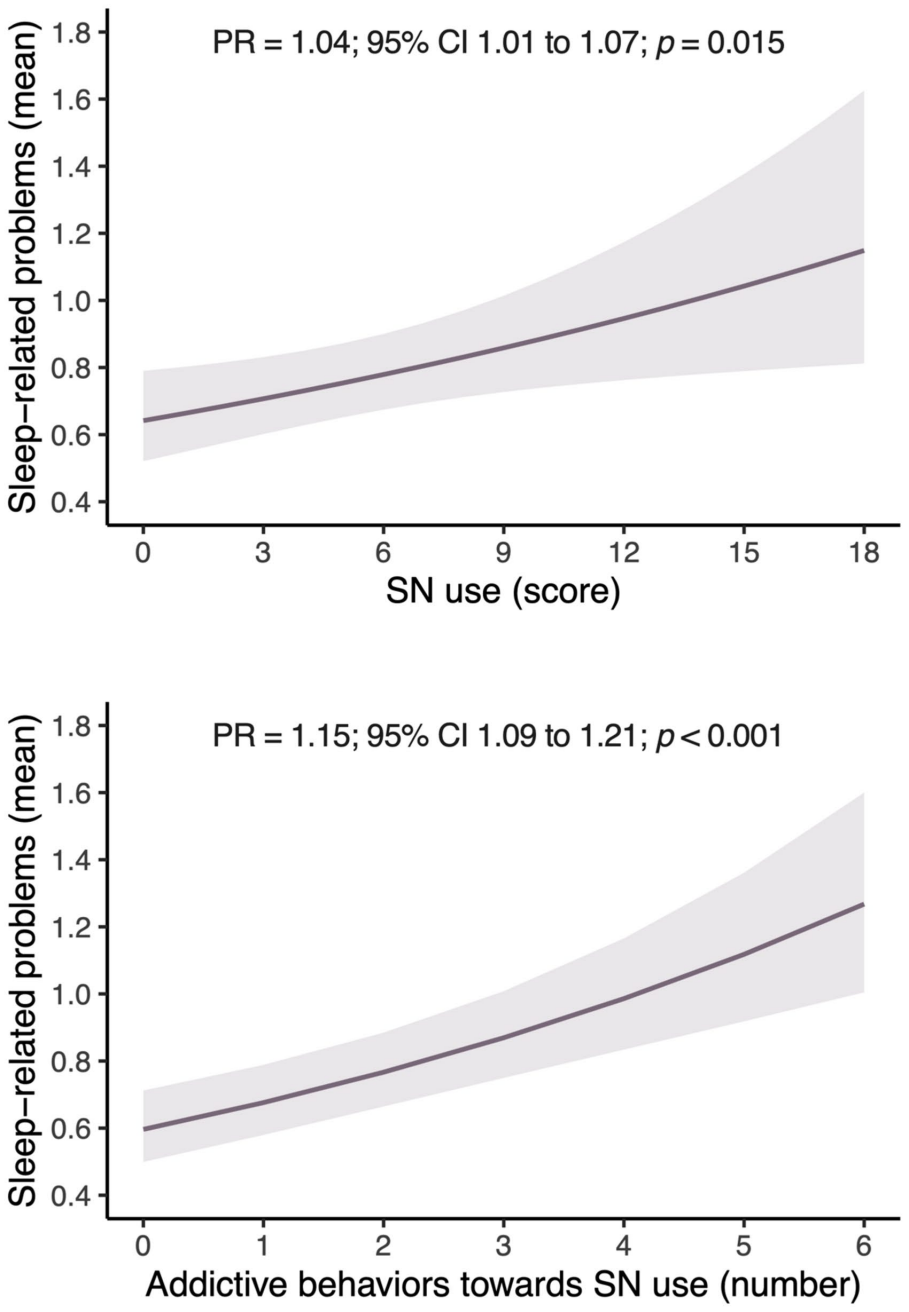
Estimated marginal means of sleep-related problems based on social network use or social network addictive behaviors in adolescents. CI, confidence interval; *p*, statistical significance; PR, prevalence ratio. SN, social network.

Figure 1 shows the predicted means of sleep-related problems in relation to SN use and the score of addictive SN behaviors in the sample of adolescents examined, after adjusting for sex, age, socioeconomic status, physical activity, sedentary behavior, BMI, and adherence to the Mediterranean diet. A positive and statistically significant association was found between higher SN use and a greater presence of sleep-related problems (PR = 1.04; 95% CI 1.01 to 1.07; *p* = 0.015). Additionally, the higher the score on the addictive behaviors toward SN use scale was, the more sleep-related problems were identified (PR = 1.15; 95% Cl 1.09 to 1.21; *p* < 0.001).

**Figure 2:**
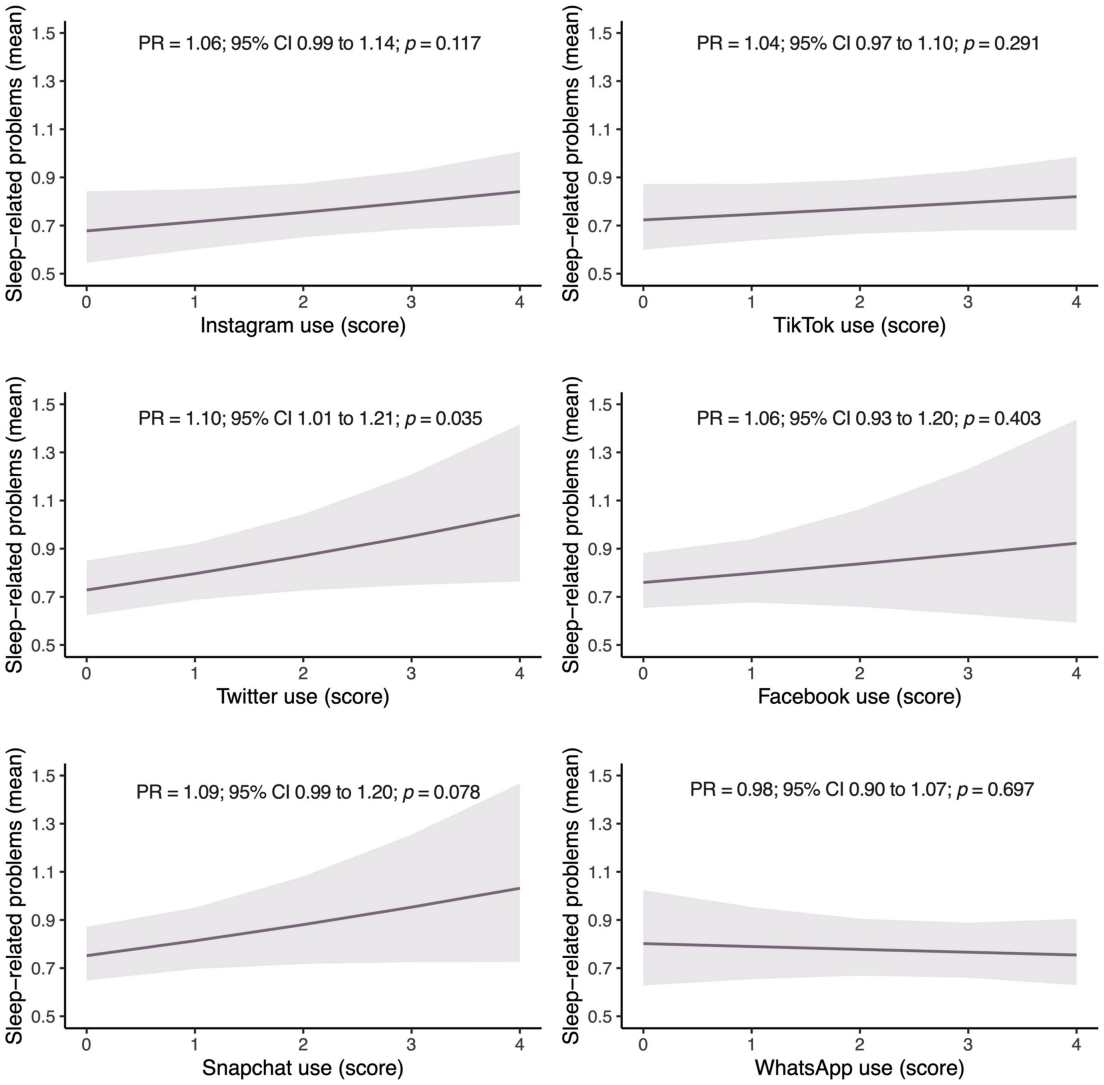
Estimated marginal means of sleep-related problems based on the use of each of the social networks in adolescents. CI, confidence interval; *p*, statistical significance; PR, prevalence ratio.

Figure 2 shows the predictive means of sleep-related problems based on the use of different SNs and a messaging application among the sample of adolescents analyzed after adjusting for the same abovementioned covariates. The use of Twitter was significantly associated with sleep-related problems (PR = 1.10; 95% Cl 1.01 to 1.21; *p* = 0.035).

**Figure 3:**
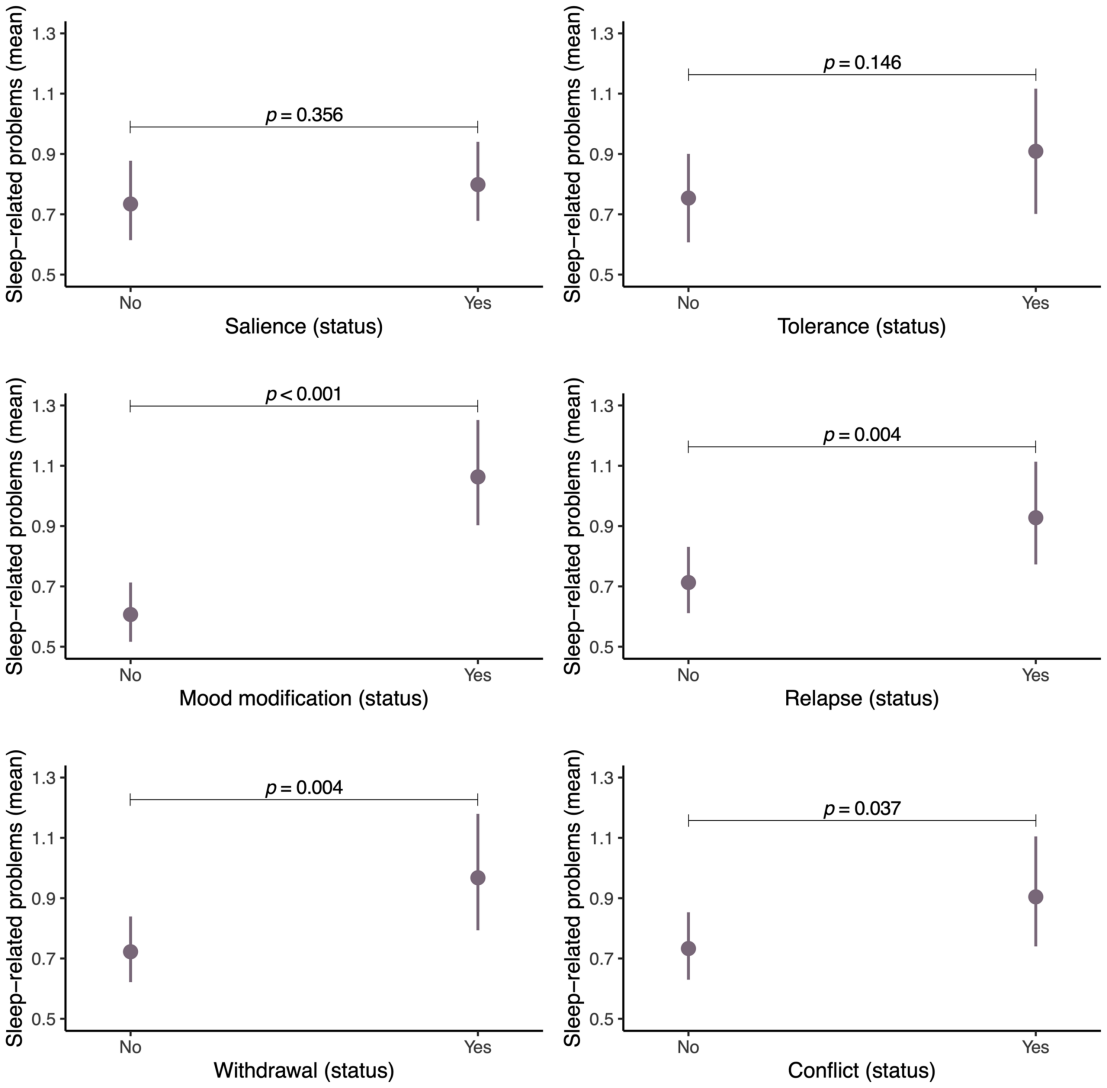
Estimated marginal means of sleep-related problems based on each sleep addictive behavior. *p*, statistical significance.

Figure 3 illustrates the predicted means and their differences for sleep-related problems on the basis of each type of addictive behavior toward SN use exhibited by adolescents, after adjusting for several covariates. Mood modification, defined as changes in mood when SNs were used, was significantly associated with sleep-related problems (PR = 1.58; 95% CI 1.36 to 1.84; *p* < 0.001). Similarly, withdrawal (the psychological and physical symptoms from not being able to use SNs), relapse (the re-emergence of usage after a period of control), and conflict (spending excessive time on SNs to the detriment of other social and everyday activities) were also associated with sleep problems (withdrawal: PR = 1.28; 95% CI 1.08 to 1.51; *p* = 0.004; relapse: PR = 1.24; 95% CI 1.07 to 1.43; *p* = 0.004; conflict: PR = 1.19; 95% CI 1.01 to 1.39; *p* = 0.037).

## 4 Discussion

To our knowledge, this study is the first to examine the link between SN use and addiction with sleep problems among Spanish adolescents, an understudied population. This is important given the rising SN usage among Spanish adolescents (Carat, 2022; Fernández, 2022) and its potential impact on mental (Seabrook et al., 2016) and physical health (Farrell et al., 2022). Our findings offer new insights into how SN use and addictive behaviors toward their use are related to sleep quality in this age phase. The results revealed a significant association between increased SN time and addictive behaviors with more sleep-related problems. Twitter was the most strongly linked to these issues, which aligns with studies indicating that some platforms have a greater impact on sleep quality than others (Scott et al., 2019; Alonzo et al., 2021), possibly because of their content update frequency, engagement type, and interaction level. Addictive behaviors such as mood changes, withdrawal, relapse, and interpersonal conflict were significantly associated with sleep issues, which is consistent with the literature connecting behavioral addictions on digital platforms to negative mental health outcomes, including disrupted sleep (Seabrook et al., 2016).

There are several potential reasons for the obtained results. First, one of the possible explanations for these findings could be related to excessive usage and disruption of sleep schedules. Adolescents who spend excessive time on social media, especially during the night, are more likely to delay bedtime (Scott and Woods, 2018). The prolonged use of electronic devices before sleep can interfere with circadian rhythms, making it harder to fall asleep and reducing sleep quality (LeBourgeois et al., 2017). Furthermore, social media addiction often leads to behaviors such as continuous scrolling, which results in a shorter sleep duration and negatively impacts overall health and well-being (Palmer et al., 2024).

Second, it is possible that adolescents with more addictive behaviors toward the use of SNs had greater exposure to stimulating content and increased emotional arousal. Social media platforms, such as Twitter (Ferrara and Yang, 2015), frequently present highly stimulating content, such as images, videos, or interactions that elicit intense emotional responses (e.g., stress, excitement, anxiety) (Schreiner et al., 2021). Prolonged exposure to such content can activate the nervous system, making it difficult for adolescents to unwind before bed (Hale and Guan, 2015; LeBourgeois et al., 2017). This emotional arousal can delay sleep onset and, in some cases, lead to disrupted sleep or nightmares, further exacerbating sleep disturbances (Vandekerckhove and Cluydts, 2010).

Third, it is possible that adolescents with more addictive behaviors toward SNs are more exposed to the reward cycle activation and dopamine release. Social media platforms are designed to maximize user engagement (Shahbaznezhad et al., 2021), triggering a reward cycle driven by dopamine release in the brain (Persson and Persson, 2023). As a consequence, the constant pursuit of “likes,” comments, or interactions can lead to addictive behaviors (Berridge and Robinson, 2016), causing adolescents to check social media even during nighttime hours (Scott et al., 2019). This ongoing search for digital rewards can keep the brain in a heightened state of alertness (Small et al., 2020), inhibiting the relaxation necessary for high-quality sleep (Carter et al., 2016).

Fourth, the notifications emitted by these devices, often accompanied by sound, can delay the onset of sleep and increase the likelihood of awakenings (Cain and Gradisar, 2010). Additionally, the light emitted by screens, particularly blue light, can have a significant effect on circadian rhythms and alertness. Evening and nighttime exposure to bright and blue light from screens can affect the timing of melatonin production, leading to physiological suppression of this sleep-promoting hormone and causing circadian disruption (Higuchi et al., 2005; Gooley et al., 2011).

The study has some limitations that must be declared. Given its cross-sectional design, causality cannot be inferred, requiring future prospective and experimental research to determine whether SN use leads to increased sleep problems. Sleep issues and SN usage were measured by a questionnaire, which adolescents may underestimate, introducing participant bias. The questionnaire focuses on SN use frequency without quantifying specific usage times, hindering the establishment of clear SN use thresholds. Although validated tools detect SN addictive behaviors, current diagnostic criteria lack a specific definition of such behaviors. Also, despite controlling for sociodemographic, lifestyle, and anthropometric factors, residual confounding factors may still influence the results. A strength of the study is the inclusion of various SNs frequently used by adolescents today. Additionally, this study provides further cross-sectional evidence on the association between SN use and sleep-related issues among Spanish adolescents.

## 5 Conclusion

The main findings of this study suggest a positive relationship between SN use and the presence of sleep-related problems in a sample of Spanish adolescents. Moreover, SN addictive behaviors are more strongly associated with sleep problems than simple SN use is. These findings add to the current growing body of evidence that increasingly addresses the physical and mental health consequences of adolescent SN use. The impairment of sleep quality due to SN use has become a public health issue and is a challenge. Childhood and adolescence are periods of vulnerability to the use of this technology, which is designed to capture as much attention and time as possible, but they are also developmental periods in which healthy habits can be learned and integrated. Therefore, health education and training in sleep hygiene and the creation of campaigns to promote the safe use of the internet and SN and to identify when there is problematic use are essential. It is necessary to develop public health projects that promote healthy relationships with SNs and the internet to improve the quality of sleep, quality of life, and health of new generations.

## Data Availability

The raw data supporting the conclusions of this article will be made available by the authors, without undue reservation.
